# Association between sleep duration and depressive symptoms among older adults in China: a national cross-sectional study

**DOI:** 10.3389/fpsyt.2026.1761122

**Published:** 2026-03-12

**Authors:** Yunting Mo, Shiyu Jiang, Jun Guo

**Affiliations:** Department of Nursing, Zhuhai Campus of Zunyi Medical University, Zhuhai, China

**Keywords:** CLHLS, cross-sectional study, depressive symptoms, older adults, sleep duration

## Abstract

**Background:**

Depression is a common mental health issue among older adults. Previous studies have suggested that sleep disturbances, including abnormal sleep duration, may be associated with an increased risk of depressive symptoms. However, the relationship between sleep duration and depressive symptoms in the context of China’s aging population remains underexplored; this study aims to investigate this association among older adults.

**Methods:**

This national cross-sectional study included 12,104 participants aged 65+ from the 2018 Chinese Longitudinal Healthy Longevity Survey (CLHLS). To assess the association between sleep duration and depressive symptoms, we used multivariate logistic regression models adjusted for sociodemographic characteristics, lifestyle factors, and comorbid chronic diseases. Additionally, trend test, restricted cubic splines (RCS) and threshold saturation effect analyses were employed to further explore the relationship between sleep duration and depressive symptoms.

**Results:**

After adjusting for confounders, increased sleep duration was linked to a lower depressive symptoms risk. The odds ratios (ORs) were 0.60 (95% CI 0.54-0.69) for 6–7 hours, 0.40 (95% CI 0.36-0.47) for 7–8 hours, 0.34 (95% CI 0.30-0.39) for 8–9 hours, and 0.32 (95% CI 0.28-0.36) for 9–15 hours. The relationship was non-linear, with an inflection point at 7 hours. Each additional hour of sleep duration was associated with a 31% (OR = 0.69, 95% CI 0.66-0.72) reduction in depressive symptoms; however, this association attenuated after 7 hours of sleep, with each additional hour corresponding to a 9% (OR = 0.91, 95% CI 0.89-0.94) reduction.

**Conclusion:**

In this nationwide survey, it is revealed that a dose - response relationship exists between sleep duration and depressive symptoms among Chinese older adults.

## 1 Introduction

Depression is a common mental health disorder among the older adults (1, 2). Systematic reviews and meta-analyses have indicated that approximately one-third of the global older adults is affected by depressive symptoms (3, 4). Depression not only leads to psychological symptoms such as low mood and decreased interest, but also may trigger physical symptoms like fatigue, insomnia, and loss of appetite, severely affecting the daily functioning and quality of life of the older adults (5). Moreover, depressive symptoms is associated with a variety of negative health outcomes, including cognitive decline, increased risk of cardiovascular disease, and suicidal tendencies (2). Studies have shown that medical expenses for patients with depressive symptoms are significantly higher than those for non-depressed individuals, and that depressive symptoms patients have higher hospitalization rates and greater demand for long-term care (2, 6, 7). With the intensification of global population aging, the prevalence of depressive symptoms among the older adults is expected to further increase, making it an increasingly important public health issue (1, 2). It is projected that by 2030, depressive symptoms will be the top cause of disease burden in middle and high-income countries (2).

Sleep is a vital physiological process for maintaining human physical and mental health (8). Sleep disorders, particularly abnormal sleep duration, have long been considered potential risk factors for depressive symptoms (9–11). Research has shown that both short sleep duration (typically defined as less than 6 hours of sleep per night) and long sleep duration (typically defined as more than 9 hours of sleep per night) are associated with adverse mental health outcomes, including an increased prevalence of depressive symptoms (12–14). Abnormal sleep duration, whether too short or too long, may increase the risk of depressive symptoms through multiple mechanisms. These mechanisms include disrupting the balance of neurotransmitter systems, such as serotonin and dopamine, which play a crucial role in emotion regulation (15–17). Additionally, changes in sleep duration may lead to circadian rhythm disruption, thereby affecting melatonin secretion. Melatonin not only regulates the sleep-wake cycle but also plays an important role in emotion regulation (18, 19). Furthermore, insufficient or excessive sleep may trigger systemic inflammatory responses, leading to increased levels of inflammatory cytokines. These cytokines may further interfere with the normal functioning of neurotransmitters, thereby exacerbating depressive symptoms (20, 21). Collectively, these mechanisms act on the emotion regulation process, thereby increasing the risk of depressive symptoms.

Given the complex relationship between sleep duration and depressive symptoms, it is essential to understand how this relationship manifests in different populations. China is experiencing accelerated population aging and faces unique challenges, particularly in addressing the mental health issues of the older adults (22). In recent years, the prevalence of depressive symptoms among the older adults in China has been on the rise. Studies have shown that the overall prevalence of depressive symptoms among older adults in Chinese nursing homes is 36.8% (23), the prevalence rate of depressive symptoms among community-dwelling older adults is 37.34% (24), and the prevalence rates of depressive symptoms among empty-nest older adults and left-behind older adults in rural areas are 34.7% (25) and 36.94% (26), respectively. These high prevalence rates place a significant strain on the public health system. However, the majority of studies on the relationship between sleep duration and depressive symptoms in the older adults have been conducted in older adults outside of China (27–32), and their findings may not be entirely applicable to the older adults in China.

China’s cultural background, sociodemographic characteristics, and lifestyle factors differ significantly from those of Western countries, and these factors may all influence the relationship between sleep and depressive symptoms. Specifically, the profile of key confounders in the Chinese older adults presents unique features that necessitate focused investigation. For instance, the current generation of Chinese older adults, especially the oldest-old, has experienced historically lower average levels of formal education compared to their Western counterparts. Studies have shown that older adults with lower levels of education may be more prone to sleep disorders and depressive symptoms (33–36). This elevated vulnerability, rooted in socioeconomic transitions, may amplify the public health impact of sleep problems in this population. Similarly, with the rapid epidemiological transition in China, the prevalence and multimorbidity patterns of chronic diseases (e.g., hypertension, diabetes) among its aging population are distinct, which may further exacerbate the risk of depression through complex physiological and psychosocial pathways (37). Given these differences and potential risks, it is particularly important to explore the specific relationship between sleep duration and depressive symptoms in the older adults in China.

This study utilized the large-scale Chinese Longitudinal Healthy Longevity Survey (CLHLS) database to investigate the relationship between sleep duration and depressive symptoms among Chinese adults aged 65 years and older. Based on existing research, we hypothesized that there is an association between sleep duration and the risk of depressive symptoms. Specifically, this study employed multivariable logistic regression models and restricted cubic spline analyses to further elucidate the relationship between the two and to assess potential threshold effects.

## 2 Methods

### 2.1 Data source

The Chinese Longitudinal Healthy Longevity Survey (CLHLS), available at https://doi.org/10.18170/DVN/WBO7LK, is a national research project initiated in 1998 by the Center for Healthy Aging and Development Studies at Peking University/National School of Development. The project employs standardized face-to-face interviews as the primary mode of data collection, wherein uniformly trained interviewers administer structured questionnaires in communities or respondents’ homes. It conducts follow-up surveys every two to three years to gather data on the health and longevity of Chinese seniors (38). The CLHLS study received approval from the Biomedical Ethics Committee of Peking University (IRB00001052–13074).

### 2.2 Study population

**Figure 1:**
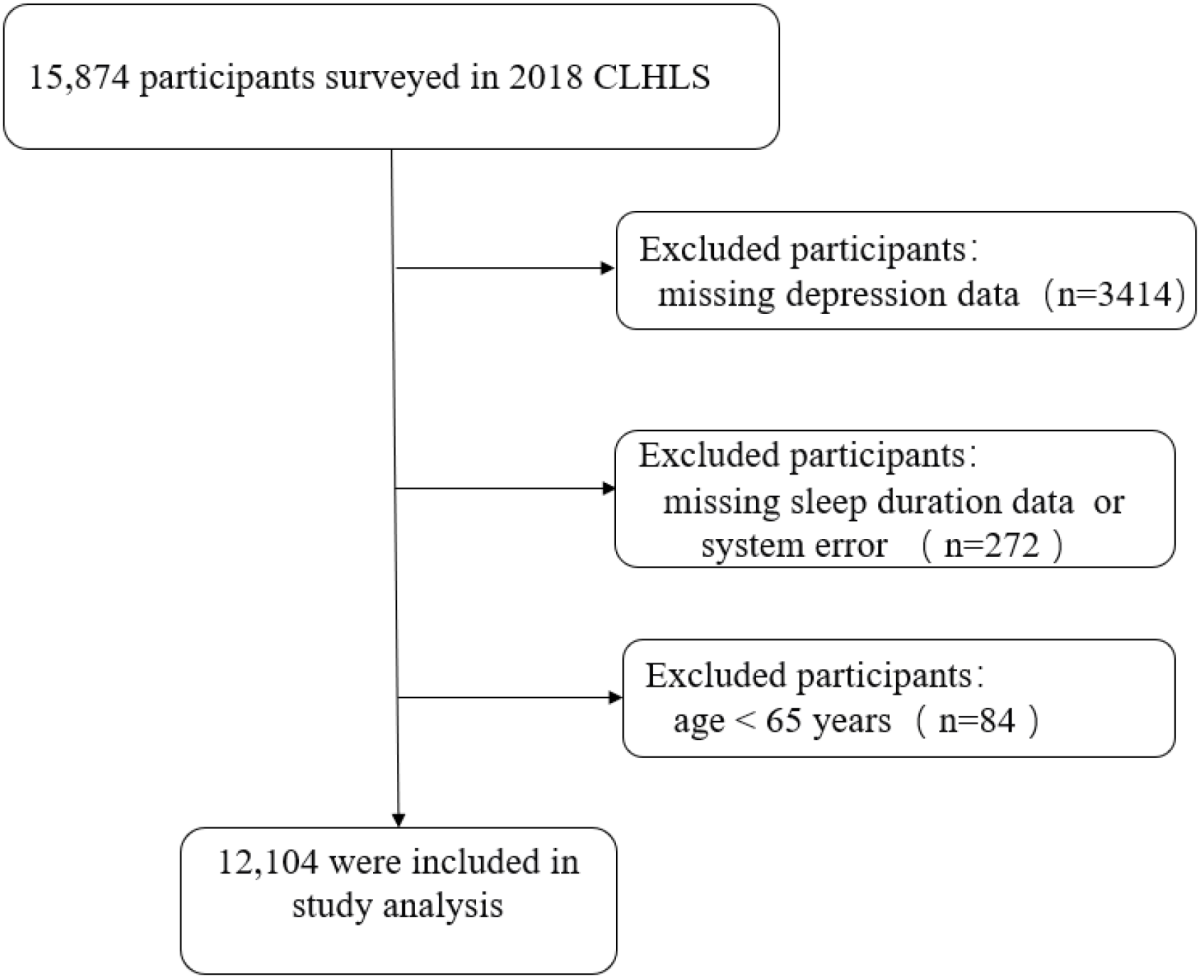
Flowchart portraying research participants.

This research utilizes data from the 2018 CLHLS for analysis. The key variables of interest, including sleep duration and depressive symptoms, were assessed via standardized, interviewer-administered questionnaires based on self-reports and did not involve proxy responses. Initially, 15,874 participants were included in the study, but 3,414 were excluded due to missing depressive symptoms data, 272 were excluded for missing sleep duration data or systemic errors, and 84 were not included due to being younger than 65 years old. Ultimately, 12,104 participants aged 65 and over were included in the study (**Figure 1**).

### 2.3 Sleep duration

Researchers used self-reporting methods to assess participants’ sleep duration (39). Participants were asked, “How many hours do you generally sleep each day now?” Participants were required to provide an integer number of hours based on their actual situation as an answer. Subsequently, researchers categorized the participants’ responses, with those who did not respond or did not know being considered as missing data.

### 2.4 Depressive symptoms

The assessment of depressive status for participants in the CLHLS utilized the 10-item Center for Epidemiological Studies Depressive symptoms Scale (CES-D-10) (40). This scale is used to evaluate depressive symptoms, including feelings of sadness, hopelessness, and depressive symptoms. It has demonstrated good reliability in studies of Chinese elderly populations, with a reported Cronbach’s α of 0.78 (40). The score range for the CES-D-10 is from 0 to 30, with higher scores indicating a more severe degree of depressive symptoms (41). In this study, referring to previous research using the CLHLS, a CES-D-10 score of ≥12 was considered to indicate the presence of depressive symptoms, while a score of <12 was regarded as the absence of depressive symptoms (42). The threshold of 12 was empirically determined as optimal for screening clinically significant depression in Chinese elderly samples, balancing sensitivity and specificity (40).

### 2.5 Covariates

Based on previous studies, we collected sociodemographic factors (age, sex, education level, household registration, marital status, and total family income in the past year), health factors (body mass index, BMI), and diagnostic conditions (hypertension, diabetes, dementia) as potential confounding factors for analysis as relevant variables (33–37). In this study, education level was categorized into four groups: less than 1 year, 1–6 years, 7–9 years, or more than 9 years. Self-reported household registration data was used to classify as rural or urban. Marital status was defined as married and living with a spouse, separated, divorced, widowed, or unmarried. The variable “Total income of household last year” is defined according to the classification in the Chinese government reports, which divides household annual income into four categories: less than 8,000 yuan($1,104), 8,000~29,999 yuan(**$**1,104~4,139), 30,000~79,999 yuan(**$**4,140~11,039), or more than 80,000 yuan($11,040). BMI was calculated by dividing weight (in kilograms) by height (in meters squared) and was based on human data measured during the CLHLS period. Diagnostic conditions included self-reported hypertension, diabetes mellitus (DM), or a history of dementia.

### 2.6 Statistical analysis

The baseline characteristics of the study participants are summarized using descriptive statistics, with continuous variables presented as mean ± standard deviation (normally distributed) or median (Q1, Q3) (non-normally distributed), and categorical variables as counts (percentages). Differences in characteristics across quintiles of the depression symptoms were tested using analysis of variance (ANOVA) or the Kruskal–Wallis test for continuous variables, depending on their distribution, and the Chi-square test for categorical variables.

Univariate and multivariate logistic regression models were constructed to assess the associations between sleep duration (independent variable) and depressive symptoms (dependent variable), with results expressed as odds ratios (ORs) and 95% confidence intervals (95% CIs). Three models were estimated: Model 1 adjusted for no covariates; Model 2 adjusted for basic demographic variables including age, sex, years of schooling, household registration, total household income in the previous year, and current marital status; and Model 3 further adjusted for additional covariates related to sleep and depression, including BMI, hypertension, diabetes, and dementia. In all models, depressive symptoms were treated as a binary outcome. Sleep duration was entered into the models both as a continuous variable and as categorical variables based on quintiles (Q1–Q5).

A test for linear trend was performed by treating the sleep duration groups (Q1–Q5) as an ordinal continuous variable (scored 1 to 5) in the logistic regression model. This test evaluates whether the odds of depressive symptoms show a statistically significant monotonic trend across the ordered categories of sleep duration. In addition, restricted cubic spline (RCS) regression was employed to explore potential nonlinear associations between sleep duration and depressive symptoms, and threshold saturation effect analysis was applied to quantify the association pattern and identify potential inflection points in the effect.

Missing data were imputed using the mice package in R, which employs multiple imputation by chained equations (MICE). All statistical analyses were performed using R (version 4.3.2). A two-sided p-value < 0.05 was considered statistically significant.

## 3 Results

### 3.1 Characteristics of study population

This study included a total of 12,104 participants with an average age of 83.34 ± 11.02 years. Of these, 46.75% were male. Sleep duration was divided into quintiles as follows: 3–6 hours, 6–7 hours, 7–8 hours, 8–9 hours, and 9–15 hours. The overall prevalence of depressive symptoms was 53.54%. Among the five quintiles of sleep duration, significant differences were observed in age, sex, education level, household registration, BMI, marital status, total household income in the past year, hypertension, diabetes, and depressive symptoms (all P < 0.001), as shown in **Table 1**.

**Table 1:** Baseline characteristics of older adults(≥65 years)(n = 12104).

Variables	Overall	Q1 3~6h	Q2 6~7h	Q3 7~8h	Q4 8~9h	Q5 9~15h	Effect size	*P*-value
	(*n* = 12,104)	(*n* =2,421)	(*n* =2,421)	(*n* =2,421)	(*n* =2,421)	(*n* =2,420)		
Age (years) ^b^	82 (74-91)	81 (73-90)	81 (72-90)	75 (71-85)	84 (78-91)	90(81-98)	0.098¹	<0.001
Sex^a^							0.087²	<0.001
Male	5,659 (46.75)	945 (39.03)	1,116 (46.10)	1,255 (51.84)	1,204 (49.73)	1,139 (47.07)		
Female	6,445 (53.25)	1,476 (60.97)	1,305 (53.90)	1,166 (48.16)	1,217 (50.27)	1,281 (52.93)		
Education ^a^							0.095²	<0.001
<1	4,577 (44.03)	1,002 (47.99)	801 (39.40)	695 (33.13)	936 (45.88)	1,143 (53.51)		
1~6	3,582 (34.46)	689 (33.00)	704 (34.63)	790 (37.65)	699 (34.26)	700 (32.77)		
7~9	1,148 (11.04)	210 (10.06)	250 (12.30)	332 (15.82)	201 (9.85)	155 (7.26)		
>9	1,088 (10.47)	187 (8.96)	278 (13.67)	281 (13.39)	204 (10.00)	138 (6.46)		
household registration^a^							0.053²	<0.001
urban	3,516 (29.09)	669 (27.66)	757 (31.31)	776 (32.08)	700 (28.96)	614 (25.45)		
rural	8,570 (70.91)	1,750 (72.34)	1,661 (68.69)	1,643 (67.92)	1,717 (71.04)	1,799 (74.55)		
BMI(kg/m^2^)^a^							0.064²	<0.001
<18.5	1,667 (14.39)	334 (14.60)	295 (12.68)	253 (10.74)	339 (14.68)	446 (19.36)		
18.5~23.9	5,993 (51.74)	1,169 (51.09)	1,184 (50.88)	1,166 (49.51)	1,226 (53.07)	1,248 (54.17)		
24.0~27.9	2,935 (25.34)	586 (25.61)	638 (27.42)	690 (29.30)	566 (24.50)	455 (19.75)		
≥28.0	989 (8.54)	199 (8.70)	210 (9.02)	246 (10.45)	179 (7.75)	155 (6.73)		
Marital status^a^							0.089³	<0.001
married	5,400 (45.08)	1,076 (44.95)	1,155 (48.35)	1,397 (58.06)	1,003 (41.74)	769 (32.20)		
separated	223 (1.86)	43 (1.80)	48 (2.01)	47 (1.95)	46 (1.91)	39 (1.63)		
divorced	45 (0.38)	14 (0.58)	9 (0.38)	10 (0.42)	4 (0.17)	8 (0.34)		
widowed	6,211 (51.84)	1,241 (51.84)	1,154 (48.30)	928 (38.57)	1,335 (55.56)	1,553 (65.03)		
never married	101 (0.84)	20 (0.84)	23 (0.96)	24 (1.00)	15 (0.62)	19 (0.80)		
Income(Yuan)^a^							0.049²	<0.001
<8000	2,868 (25.81)	722 (32.58)	555 (24.80)	513 (22.98)	550 (24.90)	528 (23.81)		
8000~29999	3,000 (27.00)	556 (25.09)	589 (26.32)	597 (26.75)	604 (27.34)	654 (29.49)		
30000~79999	2,753 (24.77)	510 (23.01)	564 (25.20)	591 (26.48)	538 (24.35)	550 (24.80)		
≥80000	2,492 (22.42)	428 (19.31)	530 (23.68)	531 (23.79)	517 (23.40)	486 (21.91)		
Hypertension^a^							0.055²	<0.001
No	6,188 (54.77)	1,137 (50.90)	1,231 (54.32)	1,226 (54.08)	1,251 (55.04)	1,343 (59.48)		
Yes	5,110 (45.23)	1,097 (49.10)	1,035 (45.68)	1,041 (45.92)	1,022 (44.96)	915 (40.52)		
DM^a^							0.066²	<0.001
No	9,634 (88.77)	1,836 (86.36)	1,901 (87.28)	1,930 (88.05)	1,948 (89.89)	2,019 (92.19)		
Yes	1,219 (11.23)	290 (13.64)	277 (12.72)	262 (11.95)	219 (10.11)	171 (7.81)		
Dementia^a^							0.030³	0.086
No	10,667(99.15)	2,082 (98.95)	2,141 (99.49)	2,161 (99.40)	2,139 (99.17)	2,144 (98.76)		
Yes	91 (0.85)	22 (1.05)	11 (0.51)	13 (0.60)	18 (0.83)	27 (1.24)		
Depressive symptoms							0.193²	<0.001
No	5,624(46.46)	713 (29.45)	1,017 (42.01)	1,301 (53.74)	1,304 (53.86)	1,289 (53.26)		
Yes	6,480(53.54)	1,708 (70.55)	1,404 (57.99)	1,120 (46.26)	1,117 (46.14)	1,131 (46.74)		

^a^Continuous variables are presented as median (Q1, Q3); ^b^Categorical variables are presented as n (%).

DM, diabetes mellitus; BMI, Body Mass Index.

Effect sizes: Cramer’s V (categorical) and ϵ² (continuous).¹ Kruskal-Wallis test; ² χ² test; ³ Monte Carlo χ² test (B = 2000).

### 3.2 Association between sleep duration and depressive symptoms

Sleep duration was divided into quintiles, and the first quintile (Q1, 3–6 hours) was used as the reference group. After adjusting for confounding factors using multivariate logistic regression analysis, the results showed that increased sleep duration was associated with a decreased risk of depressive symptoms: the odds ratios (ORs) were 0.60 (95% CI 0.54-0.69) for 6–7 hours, 0.40 (95% CI 0.36-0.47) for 7–8 hours, 0.34 (95% CI 0.30-0.39) for 8–9 hours, and 0.32 (95% CI 0.28-0.36) for 9–15 hours of sleep (**Table 2**).

**Table 2:** Association between sleep duration and depressive symptoms in multiple regression model.

Group	Model 1		Model2		Model 3	
	OR (95%CI)	*P*	OR (95%CI)	*P*	OR (95%CI)	*P*
Sleep hours						
Sleep hours as continuous variable	0.84 (0.82, 0.85)	<0.001	0.83 (0.81, 0.84)	<0.001	0.83 (0.81, 0.84)	<0.001
Q1 (3-6h)	1.0(Reference)		1.0(Reference)		1.0(Reference)	
Q2 (6-7h)	0.58 (0.51-0.65)	<0.001	0.60 (0.54-0.68)	<0.001	0.60 (0.54-0.69)	<0.001
Q3 (7-8h)	0.36 (0.32-0.40)	<0.001	0.40 (0.36-0.46)	<0.001	0.40 (0.36-0.47)	<0.001
Q4 (8-9h)	0.36 (0.32-0.40)	<0.001	0.34 (0.30-0.38)	<0.001	0.34 (0.30-0.39)	<0.001
Q5 (9-15h)	0.37 (0.33-0.41)	<0.001	0.32 (0.28-0.36)	<0.001	0.32 (0.28-0.36)	<0.001
*P* for trend	<0.001		<0.001		<0.001	

OR, odd ratio; CI, confidence interval.

Model 1: crude was not adjusted.

Model 2: age, sex, years of schooling, household registration, Current marital status, total income of household last year.

Model 3: age, sex, years of schooling, household registration, Current marital status, BMI, Total income of household last year, hypertension, DM and dementia were adjusted.

**Figure 2:**
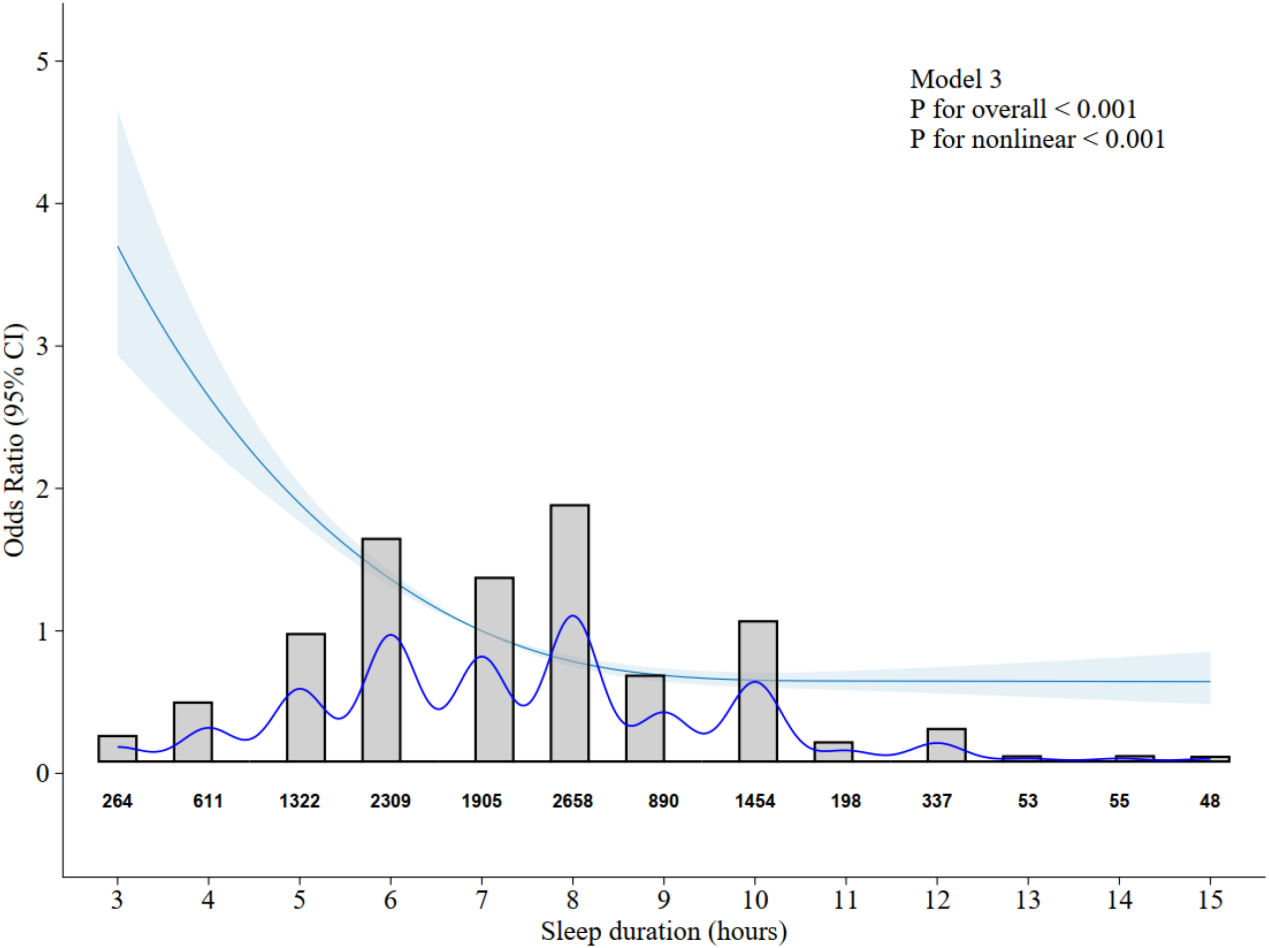
Restricted cubic spline (RCS) curve showing the association between sleep duration and odds of depression. The reference value is set at the median sleep duration (7 hours). Shaded area represents 95% confidence interval. Histogram and sample sizes below indicate the distribution of sleep duration. Age, sex, years of schooling, household registration, Current marital status, BMI, Total income of household last year, hypertension, DM and dementia were adjusted.

Restricted cubic spline suggested the relationship between sleep duration and depressive symptoms was nonlinear (P for nonlinear < 0.001; **Figure 2**). As shown in **Figure 2**; **Table 3**, the inflection point was 7 hours. Below this inflection point, each additional hour of sleep was associated with a 31% reduction in depressive symptoms risk (OR = 0.69, 95% CI 0.66-0.72). In contrast, above the inflection point, each additional hour of sleep was associated with a 9% reduction in depressive symptoms risk (OR = 0.91, 95% CI 0.89-0.94). The difference in the reduction of depressive symptoms risk before and after the inflection point was statistically significant (**Table 3**).

**Table 3:** Threshold effect analysis of sleep duration on prevalence of depressive symptoms.

Sleep duration	Adjusted OR (95% CI)	*P* value
One - line linear regression model	0.83 (0.81, 0.84)	< 0.001
Inflection point	7	
Sleep duration < 7 hours	0.69 (0.66, 0.72)	< 0.001
Sleep duration ≥ 7 hours	0.91 (0.89, 0.94)	< 0.001
Log - likelihood ratio test	< 0.001	

Adjusted for age, sex, years of schooling, household registration, current marital status, BMI, total income of household last year, hypertension, DM and dementia.

## 4 Discussion

To the best of our own knowledge, this is the first study with a large sample (n = 12,104) exploring the relationship between sleep duration and depressive symptoms among older adults in China. After adjusting for multiple potential confounding factors, we found a significant non-linear association between sleep duration and depressive symptoms. Before the inflection point (<7 hours), each additional hour of sleep duration was associated with a 31% (OR = 0.69, 95% CI 0.66-0.72) reduction in depressive symptoms; however, this association attenuated after 7 hours of sleep, with each additional hour corresponding to a 9% (OR = 0.91, 95% CI 0.89-0.94) reduction.

This study found a significant non-linear relationship between sleep duration and depressive symptoms, a finding that is consistent with the observation of several previous studies —that the relationship between the two is not simply linear— (13, 32, 43, 44), further validating the complexity of the relationship between sleep duration and depressive symptoms. However, it is noteworthy that the specific form of the non-linear association (such as the inflection point location and curve trend) may vary depending on characteristics of the study population, such as age, cultural background, and health status (45, 46). Numerous studies based on general adult or relatively younger older adult populations indicate that sleep duration and depressive symptom risk often exhibit a U-shaped or J-shaped relationship: both insufficient (e.g., <6 hours) and excessive (e.g., >9 hours) sleep durations are associated with increased depressive risk, while sleep durations around 7–8 hours are associated with the lowest risk (13, 32, 44).

Unlike the U-shaped or J-shaped non-linear associations commonly observed in previous studies, this study observed an approximate L-shaped association between sleep duration and depressive risk. Specifically, after reaching the inflection point of 7 hours, the depressive risk stabilized at a low level, and even within the longest sleep duration group (Q5, 9–15 hours, **Table 2**), no rebound in risk—as commonly seen in prior studies—was observed. We posit that this discrepancy may partly stem from the relatively limited sample size of the “very long sleep” subgroup (e.g., ≥11 hours) in this study. Among the 12,104 oldest-old individuals included in this study, only 691 individuals (**Figure 2**) reported sleep durations exceeding 10 hours, constituting a relatively low proportion. Furthermore, within the longest Q5 group (9–15 hours), the vast majority of individuals (approximately 77%) actually slept for 9–10 hours. Therefore, the overall low risk observed in this group (OR = 0.32) primarily reflects the protective effect of “longer but still within the conventional range” sleep of 9–10 hours. It is difficult to draw definitive conclusions regarding the independent association between “very long sleep” (≥11 hours) and depressive risk based on the current sample. It is also noteworthy that the individuals in the longest sleep duration group (Q5) were relatively older (see **Table 1**). Although age was adjusted for in the models, this collinearity suggests that long sleep may be associated with specific physiological states of advanced age, which should be considered when interpreting the results.

Our study identified a non-linear relationship between sleep duration and depressive symptoms in older adults, which may involve multiple physiological and psychological mechanisms. From a physiological perspective, sleep is a critical phase for bodily self-repair and regulation. During this time, the brain clears metabolic waste and regulates neurotransmitter levels (such as serotonin and dopamine), which play a crucial role in mood regulation (15–17). When sleep duration is insufficient, this self-repair and regulatory function of the brain may be disrupted, leading to neurotransmitter imbalance and thereby increasing the risk of depressive symptoms (13, 47). However, when sleep duration is adequate (e.g., reaching or exceeding the effective threshold of 7 hours found in this study), the brain’s reparative functions may be sufficiently ensured. For the older adults, longer sleep durations (e.g., 9–10 hours) may align with their specific physiological needs, representing the necessary duration for obtaining sufficient deep sleep and restoring energy, thus not exhibiting additional risk in this study. Psychological factors also play a significant role in the relationship between sleep duration and depressive symptoms (48). Adequate sleep helps older adults restore energy and enhance psychological resilience, enabling them to better cope with life stressors and challenges, thereby reducing the occurrence of depressive symptoms. Furthermore, the ability to maintain longer yet regular sleep may itself be a positive indicator of lower life stress and good physical and mental health.

The overall prevalence of depressive symptoms among the study participants (older adults) was 53.54%, which is higher than the range reported in most previous studies (no more than 40%) (23–26). We posit that this difference may primarily stem from the following reasons. First, the sample for this study was derived from the CLHLS database. Our study population is characterized by advanced average age (82 years), a high proportion of rural residents (70.91%), and a high proportion of individuals with low education levels (78.49%). These demographic features are all established risk factors for depressive symptoms (33, 34, 36). Second, the characteristics of the measurement tool and limitations in data collection must be considered. The use of the CES-D-10 scale in populations with advanced age and low education levels may involve measurement bias due to cognitive or comprehension difficulties. Moreover, as a screening scale, its results are not equivalent to a clinical diagnosis; these factors may contribute to a certain overestimation of the prevalence of depressive symptoms. Finally, this finding of a relatively high prevalence is not an isolated case. For instance, a study based on China’s CHARLS data also reported a high prevalence of depressive symptoms (over 50%) among individuals aged 60 and above (49). These pieces of evidence collectively suggest that the current older adults population in China—particularly vulnerable subgroups such as the oldest-old, rural residents, and individuals with low education levels—may be facing elevated mental health risks, urgently necessitating attention and intervention from both public health initiatives and social policies.

The findings of this study provide new evidence for understanding the unique manifestation of the relationship between sleep and depression in Chinese older adults, especially those aged 80 and above. It is worth noting that existing evidence largely comes from general adult populations in countries such as the United States, South Korea, and Japan (29, 30, 32), or from middle-aged and older adult populations in China (9, 44). Studies specifically targeting the oldest-old population (especially those aged 80 and above) in China remain scarce. Existing studies examining this relationship within Chinese populations also have certain limitations regarding the age structure of their samples. For example, the average age of participants in some studies was approximately 53 years (50), which is closer to a middle-aged population. Other studies, while confirming a non-linear relationship, did not delve into its specific form or inflection point (32). Notably, even studies employing the RCS method and explicitly focusing on older adults (43) had a sample with an average age of about 68 years and a low proportion of individuals aged 80 and above (3.24%). Given China’s current average life expectancy of 78.2 years, such samples may be insufficient in representing the rapidly growing population of the oldest-old (especially those ≥80 years). In contrast, this study, based on the Chinese Longitudinal Healthy Longevity Survey (CLHLS) database, which is specifically designed for the older adults, aims to address this research gap. Our study population has an average age of 83.34 years and includes a substantial subgroup of the oldest-old, thereby providing a more suitable sample basis for exploring the unique health status and needs of this demographic. Furthermore, compared to previous studies with limited sample sizes (e.g., n=2,959), this study included 12,104 participants. The larger sample size enhances statistical power and strengthens the stability and representativeness of the findings for the target population.

This study has several limitations. First, sleep duration was based on self-report, which may be subject to recall bias. Second, the cross-sectional design precludes the establishment of causal relationships. Third, although multiple confounders were adjusted for, control for factors such as sleep disorders, sleep quality, chronotype, substance use, and other comorbidities was not complete; therefore, the influence of residual confounding cannot be entirely ruled out. Fourth, the analytical power for the subgroup with very long sleep (≥11 hours) is limited; larger samples are needed in the future to verify its independent effect.

Despite these limitations, this study is the first to systematically reveal the unique association pattern between sleep duration and depressive symptoms among a large-scale population of the oldest-old in China. Specifically, it identifies a clear protective threshold (approximately 7 hours), beyond which no risk rebound was observed. Based on this, the study offers clear implications for practice. For the oldest-old in China, the focus of public health and clinical practice should be on ensuring they obtain at least 7 hours of adequate sleep, which should be regarded as a key, modifiable lifestyle factor for preventing depression. Simultaneously, for stable sleep patterns of 9–10 hours, provided other organic pathologies are excluded, they perhaps need not be simply pathologized or subjected to excessive intervention, a stance supported by the data from this study. However, from a general clinical prevention perspective, physicians should remain vigilant about newly emerging, excessive sleep demands (e.g., consistently exceeding 10–11 hours) to screen for potential physical or mental illnesses. This does not negate the observational findings of this study in the community-based oldest-old population but rather emphasizes the importance of individualized clinical assessment.

## 5 Conclusions

This study suggests the possibility of a non-linear relationship and threshold effect between sleep duration and depressive symptoms in older adults in China, which may provide some preliminary insights into the potential association between sleep duration and depressive symptoms in this population. Notably, our finding of a non-linear association with an inflection point around 7 hours is consistent with the established non-linear relationships and inflection point reported between sleep duration and other key geriatric outcomes like dementia (51, 52). This consistency strengthens the credibility of our finding. However, given the cross-sectional nature of the study, causality cannot be established, and the results should be interpreted with caution. Further longitudinal studies are needed to confirm these findings and explore the underlying mechanisms.

## Data Availability

Publicly available datasets were analyzed in this study. This data can be found here: https://doi.org/10.18170/DVN/WBO7LK.
